# Items

**Published:** 1901-06

**Authors:** 


### ITEMS.

The Farbenfabriken of Elberfeld Company has obtained from the United States Circuit Court of Pennsylvania, sitting in equity, a decision in its favor, the effect of which is to sustain the validity of the patent on phenacetin. If we must have patented chemical products in medicine, it is well to know that the patents are genuine, and that the original cartons are amply protected.

### SPECIAL TRAIN FOR THE AMERICAN MEDICAL ASSOCIATION.

The Chicago & Northwestern Railway has perfected arrangements to run a special train from Chicago to St. Paul to accommodate members and their friends who will attend the meeting at the latter city. The train will leave Chicago, Monday evening, June 3d, reaching St. Paul next morning in time for breakfast. The flat rate from Buffalo to St. Paul and return is $26.60.

